# Montani Semper Liberi: Age And Depression Among Veterans In Appalachia

**DOI:** 10.1093/geroni/igab046.3626

**Published:** 2021-12-17

**Authors:** Alex Dang, Maxwell Nimako, Amy Fiske

**Affiliations:** 1 West Virginia University, West Virginia University, West Virginia, United States; 2 WVU, West Virginia University, West Virginia, United States; 3 West Virginia University, Morgantown, West Virginia, United States

## Abstract

Depression is higher in rural areas and military veterans (Kimron et al., 2019; Bedard-Gilligan et al., 2018). West Virginia, the only state contained entirely within Appalachia, has a higher percentage of military service among its citizenry than other states. Thus, the purpose of the current study was to examine the association between veteran status and depression among adults in WV. Using 2018 WV data from the CDC’s Behavioral Risk Factor Surveillance System, we examined depression as a function of veteran status and age, among 612 younger adults, 1813 middle-aged adults, and 2445 older adults (N = 4,870; 12.4% veterans). Our ANOVA revealed a significant overall effect, F(5, 4864) = 14.64, p < .001, a main effect for veteran status (18.8% of veterans and 26% of non-veterans reported depression), and an age effect emerged, with more younger (28.6%) and middle-aged adults (30.5%) reporting depression than did older adults (20.3%). No significant interaction between age and veteran status emerged, F(2, 4864) = 1.75, p = .175. Of note, 25% of the sample reported having depression. Given that place-based mental health disparities exist, this finding is not unexpected. But fewer older adults and fewer veterans reported depression. At least three possibilities warrant further investigation. Future studies should examine whether these age and veteran status differences in depression reflect differences in resilience, differences in reporting, and/or differences in selective survival.

